# Detection of clinically relevant resistance genes in subgingival biofilms of chronic periodontitis: A cross-sectional molecular surveillance study

**DOI:** 10.1016/j.jobcr.2025.12.002

**Published:** 2025-12-09

**Authors:** Krishnasamy Nitya, K. Hema Shree, Aishwarya Arya, Sameep Shetty

**Affiliations:** a Department of Oral Biology, Saveetha Dental College and Hospitals, Saveetha Institute of Medical and Technical Sciences, Saveetha University, Chennai, 600077, India; b Department of Oral Biology and Oral Pathology, Saveetha Dental College and Hospitals, Saveetha Institute of Medical and Technical Sciences, Saveetha University, Chennai, 600077, India; c Department of Conservative Dentistry and Endodontics, Awadh Dental College and Hospital, Jamshedpur, Jharkhand, India; d Department of Oral and Maxillofacial Surgery, Manipal College of Dental Sciences Mangalore, Manipal Academy of Higher Education (MAHE), Manipal, India

**Keywords:** Chronic periodontitis, Antibiotic resistance genes, Subgingival biofilm, *tet(M)*, *blaTEM*, *erm(B)*, PCR, Oral resistome, India, Molecular surveillance

## Abstract

**Background:**

Antimicrobial resistance (AMR) has emerged as a major barrier to effective periodontal therapy, particularly when adjunctive antibiotics are prescribed empirically. The oral cavity—especially the subgingival biofilm—serves as a dynamic reservoir for antibiotic resistance genes (ARGs), yet data from the Indian population remain limited.

**Aim:**

To detect and characterize the presence and co-occurrence of clinically relevant ARGs—*tet(M)*, *blaTEM*, and *erm(B)*—within subgingival biofilms of Indian patients with chronic periodontitis through PCR-based molecular surveillance.

**Methods:**

This cross-sectional pilot study (n = 20) was conducted among systemically healthy adults diagnosed with chronic periodontitis (2018 AAP-EFP criteria). The sample size was pragmatically defined to assess feasibility and prevalence trends. Subgingival plaque from the deepest pocket per patient (non-pooled) was collected before instrumentation by a single calibrated examiner. DNA was extracted and subjected to end-point PCR targeting *tet(M)*, *blaTEM*, and *erm(B)*, with gene-specific positive and no-template negative controls included in each run. Amplification reproducibility was verified through cross-laboratory concordance testing. Descriptive and Fisher's exact analyses were applied to explore gender-wise patterns.

**Results:**

*tet(M)* was detected in 65 % of patients, *blaTEM* in 45 %, and *erm(B)* in 30 %. Dual-gene co-occurrence was observed in 35 %, while 20 % harbored all three genes. The most frequent association was *tet(M)* + *blaTEM*. Males exhibited a higher mean gene burden (1.8 ± 0.4) than females (1.4 ± 0.5), though differences were not statistically significant (*p* > 0.05).

**Conclusion:**

This pilot demonstrates a substantial prevalence and co-occurrence of resistance determinants in periodontal biofilms, underscoring the oral cavity's role in local and systemic AMR propagation. While preliminary, these findings support incorporating molecular resistance surveillance into periodontal diagnostics and tailoring region-specific antibiotic stewardship strategies to preserve therapeutic efficacy.

## 1 Introduction

Periodontitis is a chronic inflammatory disease of the supporting structures of the teeth, driven by a complex interplay between subgingival biofilms and host immune responses.^1^ While mechanical debridement remains the cornerstone of periodontal therapy, systemic antibiotics are often prescribed as adjuncts in aggressive or refractory cases to suppress pathogenic microbial populations.^2^^,^^3^ However, the growing threat of antimicrobial resistance (AMR) has raised significant concerns regarding the indiscriminate use of antibiotics in dental practice.^4^

The human oral cavity harbors a diverse and densely populated microbial ecosystem, with subgingival plaque offering a protected niche for the development and persistence of antibiotic-resistant organisms. Biofilms, by their nature, facilitate horizontal gene transfer and protect resident microbes from antimicrobial agents, making them ideal reservoirs for antibiotic resistance genes (ARGs). Of particular relevance in periodontal infections are resistance determinants against commonly prescribed antibiotic classes, including tetracyclines, beta-lactams, and macrolides.^5^^,^^6^

Among the most widely studied ARGs in the oral microbiota are *tet(M)*, which confers resistance to tetracyclines via ribosomal protection proteins; *blaTEM*, which encodes TEM-type beta-lactamases capable of hydrolyzing penicillin derivatives; and *erm(B)*, which methylates 23S rRNA, thereby conferring resistance to macrolides. The detection of these genes in subgingival samples can have direct clinical implications, as they may compromise the efficacy of empiric antibiotic regimens in periodontal therapy.^7^^,^^8^

Understanding the prevalence and co-occurrence of these determinants in the Indian subgingival microbiome can inform region-specific antibiotic stewardship and guide molecular surveillance strategies.

Despite increasing global attention to AMR, there is a paucity of molecular surveillance data on the prevalence of these resistance genes within periodontal niches in the Indian population. Most existing studies have been conducted in Western or East Asian settings, and data from South Asia remain limited. This pilot study aims to bridge this gap by evaluating the prevalence and co-occurrence of *tet(M)*, *blaTEM*, and *erm(B)* genes in the subgingival plaque of Indian patients diagnosed with chronic periodontitis. Understanding the local resistome may inform more judicious use of antimicrobials in periodontal care and highlight the need for incorporating resistance profiling into routine clinical practice.

Given the pilot nature of this study, the findings are intended to provide baseline prevalence data and assess the feasibility of broader multicentric surveillance.

## 2 Materials and methods

### 2.1 Study design and ethical clearance

This cross-sectional pilot study was conducted over a two-month period in the Department of Periodontology. The study protocol was reviewed and approved by the Institutional Ethics Committee (Approval No: ADCH/2024/124). All participants provided written informed consent prior to inclusion in the study. The design adhered to the ethical principles outlined in the Declaration of Helsinki.

Given the pilot nature of this study, the sample size (n = 20) was determined pragmatically based on feasibility, time constraints, and resource availability rather than formal power estimation. The primary objective was to assess the feasibility of detecting ARGs and to generate preliminary prevalence data to inform larger multicentric studies.

### 2.2 Study population

The study population comprised patients diagnosed with chronic periodontitis who attended the outpatient department. Inclusion criteria were as follows: patients aged between 18 and 60 years, diagnosed with chronic periodontitis according to the 2018 classification by the American Academy of Periodontology and the European Federation of Periodontology (AAP-EFP), with at least four periodontal sites exhibiting probing depth (PD) of ≥5 mm and clinical attachment loss (CAL) of ≥2 mm. Patients were required to be systemically healthy and must not have taken antibiotics in the past three months.

Exclusion criteria included tobacco use (smoking or smokeless forms), pregnancy or lactation, history of periodontal therapy within the preceding three months, and systemic immunosuppressive conditions. Tobacco users were excluded to minimize a major confounding factor, as smoking significantly alters the subgingival microbiome and may influence antibiotic resistance gene carriage**.**

A total of 20 eligible patients meeting the criteria were consecutively recruited for this study.

### 2.3 Sample collection procedure

Following routine clinical examination and site selection, supragingival plaque was removed using sterile gauze and cotton rolls to minimize contamination. Subgingival plaque samples were collected from the single deepest periodontal pocket of each patient (one site per patient; samples were not pooled) using sterile Gracey curettes**.** Sampling was performed by a single calibrated examiner to ensure consistency (intra-examiner reproducibility >90 % for PD/CAL measurements).

The collected plaque was immediately transferred into sterile microcentrifuge tubes containing 1 mL of TE buffer (10 mM Tris-HCl, 1 mM EDTA, pH 8.0). Samples were properly labeled and stored at −20 °C until DNA extraction was performed.

Subgingival plaque was collected from the deepest periodontal pocket of a mandibular first molar (tooth #36 or #46), as this site is frequently affected in chronic periodontitis and offers a standardized, anaerobic microenvironment representative of subgingival biofilm communities used for microbial and resistance gene analysis [Kumar PS et al., 2011].^9^

### 2.4 DNA extraction

Genomic DNA was extracted using the QIAamp DNA Mini Kit (Qiagen, Germany) following the manufacturer's protocol. The concentration and purity of extracted DNA were measured using a NanoDrop spectrophotometer (Thermo Scientific, USA), with absorbance ratios at 260/280 nm used to assess purity. Only samples with A260/280 values between 1.7 and 2.0 were deemed suitable for downstream molecular analysis.

### 2.5 PCR-based detection of resistance genes

The presence of three antibiotic resistance genes — *tet(M)*, *blaTEM*, and *erm(B)* — was assessed using polymerase chain reaction (PCR). The specific primers used for each gene were as follows:

*tet(M)* (Forward: GTGGACAAAGGTACAACGAG, Reverse: CGGTAAAGTTCGTCACACAC, 406 bp product),

*blaTEM* (Forward: ATGAGTATTCAACATTTCCG, Reverse: CCAATGCTTAATCAGTGAGG, 850 bp product), and

*erm(B)* (Forward: GAAAAGGTACTCAACCAAATA, Reverse: AGTAACGGTACTTAAATTGTTTAC, 639 bp product).

All primers were commercially synthesized (Bio-Rad, USA).

PCR reactions were carried out in 25 μL volumes containing 12.5 μL of 2X PCR Master Mix (Taq polymerase, dNTPs, buffer), 1.0 μL of each primer (10 μM), 2.0 μL of template DNA, and 8.5 μL of nuclease-free water. Thermal cycling conditions were standardized for 35 cycles as follows: initial denaturation at 95 °C for 5 min; denaturation at 95 °C for 30 s; annealing at optimized temperatures specific to each primer set (55 °C for *tet(M)*, 56 °C for *blaTEM*, and 57 °C for *erm(B)*) for 30 s; extension at 72 °C for 1 min; and a final extension step at 72 °C for 10 min.^9^

**Quality control measures:** Each PCR run included no-template negative controls and gene-specific positive controls (*Enterococcus faecalis* for *tet(M)*, *Escherichia coli* for *blaTEM*, and *Enterococcus faecalis* for *erm(B)*). All runs were validated only when both positive amplification and negative control absence were confirmed.

### 2.6 PCR result confirmation and interpretation

Due to logistical constraints, in-house agarose gel visualization was not performed. Instead, amplification confirmation relied on reproducible, target-specific amplification under standardized PCR conditions**.** Gene presence was determined by consistent amplification signals, verified through replicate assays.

A subset of samples was cross-validated in a collaborating laboratory equipped with UV documentation systems for gel electrophoresis, confirming expected amplicon sizes and reproducibility.

The lack of on-site gel documentation is acknowledged as a methodological limitation, addressed through inter-laboratory verification to ensure reliability.

### 2.7 Data management and analysis

All PCR results were recorded as binary variables, indicating the presence or absence of each resistance gene per sample. Descriptive statistics were used to calculate gene-specific frequencies and percentages. A resistance gene burden score was assigned to each sample based on the total number of detected genes (range: 0–3).

A co-occurrence matrix was constructed to identify patterns of multiple gene detection within individual samples.

Gender-wise comparisons of gene prevalence were evaluated using Fisher's exact test due to small subgroup sizes. Two-sided ***p*** < 0.05 was considered significant. These analyses were exploratory and intended to generate hypotheses for larger studies.

Data were compiled and analyzed using Microsoft Excel 365 and IBM SPSS Statistics for Windows, Version 26.0 (IBM Corp., Armonk, NY).

## 3 Results

### 3.1 Overview of key findings

In this pilot investigation involving 20 systemically healthy patients with chronic periodontitis, the presence of three clinically significant antibiotic resistance genes—*tet(M)*, *blaTEM*, and *erm(B)*—was assessed in subgingival plaque samples using PCR-based molecular detection. The PCR amplification signals were verified through replicate reactions and subset cross-validation in an external laboratory, ensuring reproducibility despite the absence of in-house gel visualization.

**Fig. 1:**
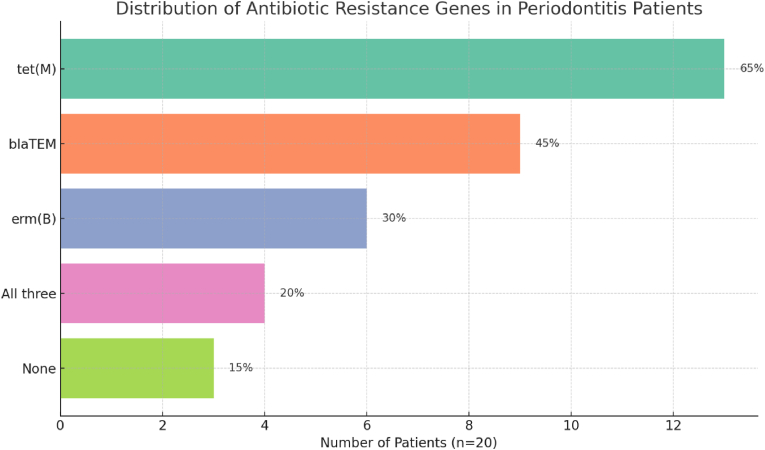
Horizontal bar graph showing distribution of antibiotic resistance gene detection in chronic periodontitis patients.

The results revealed that *tet(M)* was the most prevalent resistance gene, detected in 65 % of the samples, followed by *blaTEM* in 45 % and *erm(B)* in 30 %. Notably, 20 % of patients harbored all three resistance genes, while only 15 % were negative for all tested genes, indicating a broad dissemination of resistance determinants within the subgingival biofilm, even in patients without recent antibiotic exposure (Table 1, Fig. 1).

**Table 1:** Prevalence of antibiotic resistance genes in subgingival plaque (n = 20).

Gene	Positive Samples (n)	Prevalence (%)
*tet(M)*	13	65 %
*blaTEM*	9	45 %
*erm(B)*	6	30 %
All three	4	20 %
None	3	15 %

### 3.2 Detailed interpretation of individual genes

*tet(M)* emerged as the most frequently detected resistance gene, found in 13 of 20 patients (65 %). This gene encodes a ribosomal protection protein that mediates resistance to tetracyclines—antibiotics commonly used in periodontal therapy, especially for refractory or aggressive forms. The high detection rate aligns with previously reported data from European and Asian cohorts and likely reflects past tetracycline exposure, selective pressure within oral biofilms, and potential horizontal gene transfer.

The *blaTEM* gene, encoding TEM-type beta-lactamases, was present in 45 % of samples. Given the frequent empirical use of beta-lactams such as amoxicillin in dental practice, the presence of *blaTEM* suggests emerging subgingival microbial resistance with potential to compromise treatment efficacy.

Detection of *erm(B)*, a 23S rRNA methylase gene conferring macrolide resistance, was observed in 30 % of cases. Though less common, its occurrence underscores the latent threat to macrolide-based alternatives such as erythromycin and azithromycin. The lower frequency may reflect reduced macrolide exposure in standard dental regimens, but its co-existence with other genes implies active horizontal gene exchange among oral bacteria.

### 3.3 Co-occurrence and gene burden

**Fig. 2:**
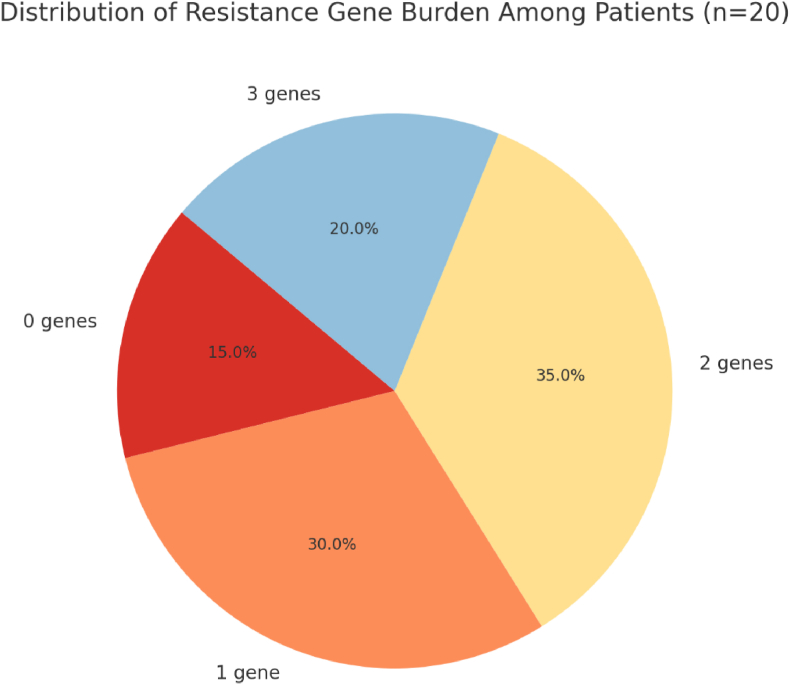
Pie chart showing distribution of antibiotic resistance gene burden among study participants.

Co-occurrence analysis revealed that 35 % of patients harbored two resistance genes and 20 % carried all three. The **mean resistance gene burden per patient was 1.6 ± 0.87**, reflecting the presence of multiple ARGs within individual subgingival niches (Table 2, Fig. 2).

**Table 2:** Antibiotic resistance gene burden per patient.

Number of Genes Detected	Patients (n)	Percentage (%)
0	3	15 %
1	6	30 %
2	7	35 %
3	4	20 %

**Fig. 3:**
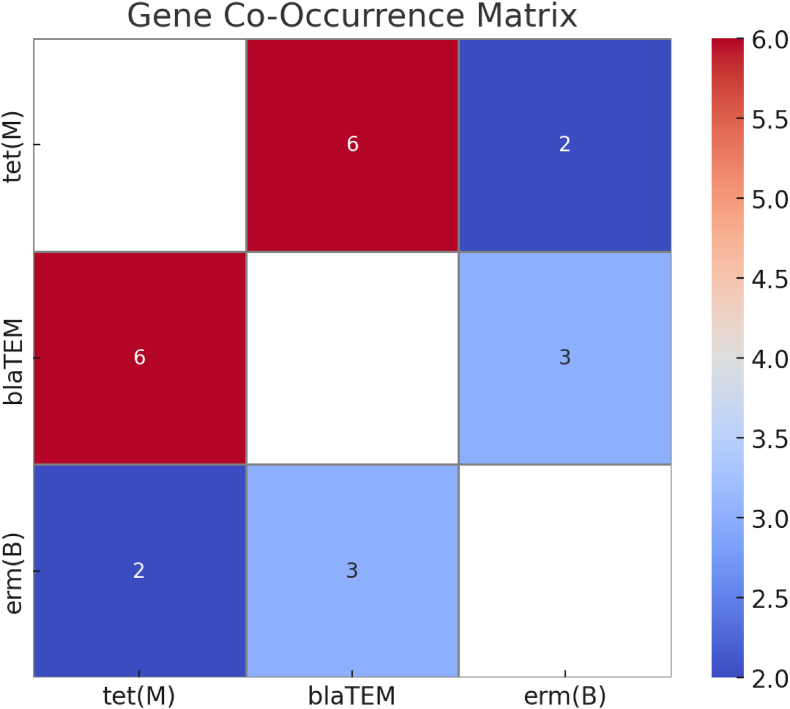
Heatmap showing co-occurrence patterns among resistance genes detected in subgingival plaque samples.

A co-occurrence matrix demonstrated *tet(M)* + *blaTEM* as the most frequent combination (n = 6), followed by *blaTEM* + *erm(B)* (n = 3) (Table 3, Fig. 3).

**Table 3:** Co-occurrence matrix of resistance genes.

	tet(M)	blaTEM	erm(B)
*tet(M)*	–	6	2
*blaTEM*	6	–	3
*erm(B)*	2	3	–

### 3.4 Biological and clinical implications

The detection of multiple antibiotic resistance genes within subgingival biofilms reinforces the concept of the oral cavity as an active microbial reservoir for antimicrobial resistance. Biofilm-mediated protection and gene exchange mechanisms enable stable maintenance of multi-resistance profiles.

These findings have direct clinical relevance, as the uncritical use of systemic antibiotics in periodontal therapy—without phenotypic verification—can select for resistant bacterial subpopulations. It is essential to interpret these molecular results cautiously, since the detection of ARGs does not confirm phenotypic resistance. Functional assays (e.g., MIC testing or metatranscriptomic profiling) are required to confirm clinical relevance.

Nevertheless, the results emphasize the need for antibiotic stewardship programs in periodontology and suggest that molecular resistance screening may assist in managing refractory or recurrent cases.

### 3.5 Gender-based trends (exploratory analysis)

**Fig. 4:**
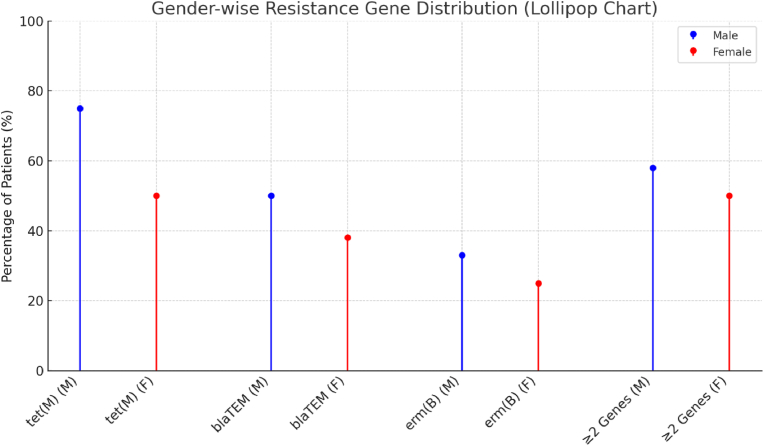
Lollipop plot illustrating gender-wise distribution and co-occurrence of antibiotic resistance genes.

An exploratory comparison revealed a higher mean resistance gene burden among males (1.8 ± 0.4) than females (1.4 ± 0.5), though the difference was not statistically significant **(**Fisher's exact test, p > 0.05). Among male participants, *tet(M)* was detected in 75 %, *blaTEM* in 50 %, and *erm(B)* in 33 %, while among females, detection rates were 50 %, 38 %, and 25 %, respectively (Table 4, Fig. 4).

**Table 4:** Gender-wise distribution of resistance genes.

Gender	n	*tet(M)*	*blaTEM*	*erm(B)*	≥2 Genes (%)
Male	12	9 (75 %)	6 (50 %)	4 (33 %)	7 (58 %)
Female	8	4 (50 %)	3 (38 %)	2 (25 %)	4 (50 %)

These subgroup findings are descriptive and should be interpreted cautiously due to the small sample size and pilot design.

## 4 Discussion

The findings of this pilot study provide compelling evidence that antibiotic resistance genes (ARGs) are frequently present in the subgingival biofilms of patients with chronic periodontitis. Among the three clinically relevant genes evaluated, *tet(M)* was the most frequently detected (65 %), followed by *blaTEM* (45 %) and *erm(B)* (30 %). Furthermore, a significant proportion of patients (35 %) carried two resistance genes, and 20 % harbored all three, suggesting a notable burden of multidrug-resistance potential within the periodontal microbiota. These results indicate that the subgingival environment may act as a persistent microbial reservoir of resistance determinants**,** even in systemically healthy individuals with no recent antibiotic exposure.

Our observations are consistent with previous reports from other geographic regions. Rams et al. (2021) documented comparable prevalence rates of *tet* and *blaTEM* genes in chronic periodontitis samples from the United States, while Ready et al. (2015) detected *erm* and *tet* markers in oral anaerobes such as *Porphyromonas gingivalis* and *Tannerella forsythia*.^10^^,^^11^ These parallels underscore the global significance of the oral resistome and the necessity of sustained molecular surveillance. The prevalence figures observed in our Indian cohort also reflect the broader trends of antimicrobial resistance driven by unregulated antibiotic consumption and highlight the urgent need to strengthen antibiotic-stewardship practices in dental settings.

The detection of *blaTEM* in nearly half the participants is particularly concerning given the routine empirical use of beta-lactam antibiotics such as amoxicillin in periodontal therapy. Resistance to these agents could reduce treatment efficacy, especially in severe or refractory disease. Likewise, the presence of *erm(B)* in nearly one-third of cases signals a potential emerging threat to macrolide efficacy, although its comparatively lower frequency may reflect the less frequent use of macrolides in dentistry. The high cumulative gene burden in several patients suggests horizontal gene transfer and stable incorporation of resistance elements within the oral biofilm, further complicating antimicrobial management.^12^

While numerous studies corroborate these results, others have reported lower detection frequencies of ARGs in subgingival plaque. For instance, Nibali et al. (2020) observed a relatively low prevalence of resistance genes in untreated periodontitis patients from Norway, possibly due to regional differences in prescribing habits, microbial ecology, or host factors. Such contrasts highlight the influence of geographic and clinical context on resistome composition and reaffirm the need for region-specific surveillance rather than extrapolation from international data.^13^

The broader implications of these findings extend beyond periodontal practice. The subgingival niche—characterized by dense multispecies biofilms and continuous exposure to host immune mediators—provides optimal conditions for gene exchange and persistence of resistance determinants**.** The detection of ARGs within this environment suggests that the oral cavity could play a role in the community- and system-level dissemination of antimicrobial resistance. This aligns with the *One Health* framework, which emphasizes the interdependence of oral, systemic, and environmental health in combating the global AMR crisis.^14^^,^^15^

An exploratory gender-based comparison revealed a slightly higher resistance gene burden among males than females. Although not statistically significant, this trend may reflect differences in oral hygiene behavior, prior antibiotic exposure, or disease severity. Given the limited sample size, these demographic patterns should be regarded as hypothesis-generating and warrant validation in larger, adequately powered cohorts.

In light of these observations, the study advocates a shift toward rational and evidence-based antibiotic prescribing in periodontology**.** Incorporating molecular resistance profiling could aid in the management of non-responding or recurrent cases by guiding the selection of more effective, targeted regimens. Furthermore, these findings strengthen the argument for integrating antimicrobial-resistance awareness into dental curricula, clinical protocols, and continuing education, in line with global stewardship initiatives.

## 5 Limitations

This study is limited by its small sample size (n = 20), which constrains statistical power and generalizability. The use of conventional endpoint PCR restricted analysis to the mere detection of ARGs without assessing their expression levels or copy number variations. Additionally, the study did not incorporate species-level microbial identification**,** precluding attribution of ARGs to specific periodontal pathogens.

The absence of in-house agarose gel electrophoresis, though mitigated by cross-validation of a subset in an external laboratory, remains a methodological limitation.

Finally, the lack of longitudinal follow-up prevents correlating gene presence with treatment outcomes or disease progression.

## 6 Future perspectives

Future studies should include larger, multicentric cohorts with healthy controls to enable comparative analysis and enhance external validity. Employing quantitative or digital PCR would provide insights into gene expression dynamics and relative resistance load. Integration of 16S rRNA or metagenomic sequencing could elucidate the microbial hosts of ARGs and their network interactions. Longitudinal designs evaluating resistome shifts before and after periodontal therapy would clarify the therapeutic impact on resistance profiles.

Overall, embedding molecular surveillance into routine periodontal diagnostics can improve antibiotic stewardship and guide more personalized, effective, and sustainable treatment strategies.

## 7 Conclusion

This pilot study highlights the detection of clinically relevant antibiotic resistance genes—*tet(M)*, *blaTEM*, and *erm(B)*—within subgingival biofilms of patients with chronic periodontitis. The frequent co-occurrence of these genes indicates that the oral cavity may act as a hidden reservoir of antimicrobial resistance with implications for both local and systemic health. These findings emphasize the importance of region-specific molecular monitoring and judicious antibiotic use to preserve therapeutic efficacy. As antimicrobial resistance continues to escalate globally**,** integrating resistance surveillance and stewardship principles into routine dental practice is essential for sustaining clinical outcomes and public health.

## Patient consent

Patient/guardian consent was obtained.

## Ethical clearance

Ethical clearance was obtained from the Institutional Review Board.

## Source of funding

Self-funded project.

## Declaration of competing interest

The authors declare that there is no conflict of interest.
